# Hydrodynamic independence and passive control application of twist and flapwise deformations of tidal turbine blades

**DOI:** 10.1016/j.jfluidstructs.2022.103827

**Published:** 2023-04

**Authors:** Federico Zilic de Arcos, Christopher R. Vogel, Richard H.J. Willden

**Affiliations:** University of Oxford. Department of Engineering Science, Parks Rd., OX1 3PJ, Oxford, UK

**Keywords:** Hydrodynamics, Axial-flow turbine, Fluid–structure interaction, Passive turbine control, Tidal energy

## Abstract

The load-induced deformations experienced by axial-flow rotor blades can result in significant hydrodynamic impacts on rotor operation. These changes in hydrodynamics are dominated by the flapwise and twist deformation components, affecting blade loading and performance. This work uses blade-resolved computational fluid dynamics simulations to explore the hydrodynamic interactions of coupled flapwise and twist deformations, and their potential for use in passive control strategies. The rotor blades were simulated under parametrically prescribed flapwise-only, twist-only and coupled flapwise–twist deformations. The results show that the hydrodynamic effects are adequately described by blade-element theory for twist deformations regardless of the presence of flapwise deformations, whereas flapwise deformations induce changes in the local lift and drag coefficients that are independent of twist. For moderate blade deflections, the hydrodynamic changes generated by the two deformation components can be approximated to be independent from each other. The observed hydrodynamic independence between the two deformation components is used to explore passive deformation strategies for a tidal rotor. By extrapolating an existing dataset containing CFD simulations of twist-only and flapwise-only deformation cases at different tip-speed ratios, control paths are designed within a tip-speed ratio, flapwise and twist deformation parameter space. These control paths demonstrate passive control strategies as a potential alternative to active pitch control on tidal turbines, showing similar performance and maximum loading, compared with an active pitch strategy, over a full tidal cycle. In particular, it is shown that flapwise deformations have an important role in power capping above rated flow speed.

## 1 Introduction

Tidal streams are an abundant and renewable energy resource which is highly predictable, offering an advantage over other, more intermittent, renewables such as photovoltaic and wind. However, converting tidal stream energy into electricity at utility scale remains challenging due to the technological and operational complexities of the marine environment. Significant progress has been observed in recent years for tidal stream energy, with megawatt-scale devices at pre-commercial stages deployed and connected to electricity grids (e.g. Orbital Marine’s O2 (Orbital Marine Power, 2021), Simec Atlantis AR1500 (Atlantis Resources, 2016), Hammerfest Strøm HS1000 (Andritz Hydro Hammerfest, 2015)). Despite these advances, efforts are still required to reduce the Levelised Cost of Energy (LCOE) for this energy resource to be competitive at a utility scale, for which significant technological improvements are still required (Smart and Noonan, 2018).

Despite outward similarities between wind and tidal stream turbines, a number of differentiating factors exist, such as blockage (Schluntz and Willden, 2015, Zilic de Arcos et al., 2020a), cavitation (Wimshurst et al., 2018), or exposure to transient flow effects such as turbulence, shear, and waves (Milne et al., 2016, Wimshurst and Willden, 2016, Ordonez-Sanchez et al., 2019). Consequently, concepts transferred from the wind sector should be assessed to ensure that the relevant physics in the tidal environment is captured and to support the development of improved modelling tools which are required to devise optimised solutions.

Turbine control strategies are one such important element to reassess. Large-scale wind rotor successfully operate with active blade-pitching systems. Such systems, integrated with adequate control strategies, contribute to reduce LCOE despite the rotor’s increased mechanical complexity through the improved aerodynamic performance of the rotor. However, due to the logistical constraints imposed by the marine environment, the reliability of blade-pitching systems underwater have been identified as one of the most critical components affecting the availability (and, thus, revenue) of tidal stream energy converters (Le Diagon et al., 2021). Thus, devices of reduced mechanical complexity, with lower failure rates and maintenance requirements, might be a more suitable alternative.

A potential alternative is implementing passive turbine control, a concept that relies on rotor blade deformations to achieve control objectives and that stems from the observation that blade deformations, driven by hydrodynamic loads and complex fluid–structure interactions, can significantly influence rotor loads and/or performance.

**Fig. 1:**
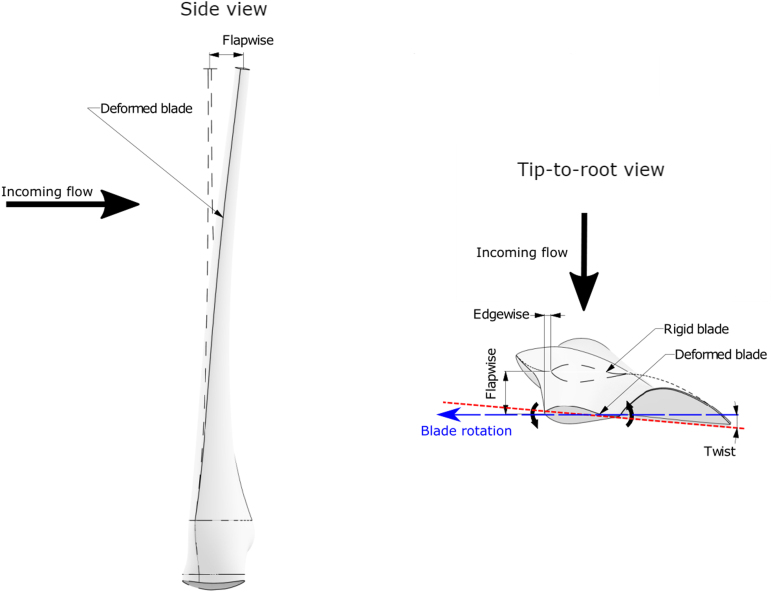
Deformation degrees of freedom for a turbine blade as seen from side (left) and blade tip-to-root (right) views. The two views show a three-dimensional render of the deformed blade alongside an outline of the undeformed blade. The nacelle and the remaining blades have been omitted for clarity.

Rotor blade deformations are typically separated into three main components: flapwise, edgewise and twist, illustrated in Fig. 1. The three deformation components are caused by, respectively: the thrust force; the tangential force that drives the rotation of the blades; and the torsional moment created by the offset between the hydrodynamic centre of pressure and the structural centre, as well as from bending–twist structural couplings (Fedorov and Berggreen, 2014). The magnitude of blade deflections can be significant for the flapwise and twist components, especially when blades are designed using flexible composite materials. Flapwise deformations, for example, can reach values of up to 10%–15% of the turbine radius while operating at maximum flow speeds (see e.g. Nicholls-Lee (2011), Grogan et al. (2013) and Rafiee et al. (2016)), whilst twist deformations have been reported to reach values of approximately 2.5° to 5.0° at the tip when anisotropic composite materials are used (see e.g. Ostachowicz et al. (2016) Murray et al. (2016), De Goeij et al. (1999), Lobitz and Veers (2003)). Edgewise deformations, in contrast, are of lesser importance because they are caused by tangential forces, typically an order of magnitude smaller than the axial forces and oriented in a direction where blade stiffness tends to be higher (Locke et al., 2003, Zilic de Arcos et al., 2018).

The exploitation of the blade structure to obtain a desired hydrodynamic response requires a detailed knowledge of the loading as well as of the structural response. This fluid–structure interaction (FSI) problem is complex, however, because the deformation of the blades affects the hydrodynamic loads, which in turn affect blade deflections.

FSI phenomena on wind and tidal rotors have been investigated using methods of varying complexity. Such models are typically developed by integrating structural and fluid dynamics solvers. For fluid dynamics modelling, blade-element momentum (BEM) type models (see e.g. Zilic de Arcos et al. (2018), Murray et al. (2016)), potential flow solvers (Nicholls-Lee (2011)) and blade-resolved unsteady CFD computations (Bazilevs et al. (2011), Grinderslev et al. (2021)) have been used. On the structural side, FSI models typically use finite element solvers with either beam (Jonkman, 2013, Larsen and Hansen, 2007) or shell elements (Zilic de Arcos et al., 2018, Nicholls-Lee, 2011).

Tightly-coupled high-fidelity FSI models, capable of capturing the hydrodynamics of blade deformations as well as the structural dynamics, tend to have a prohibitively high computational cost for engineering design and optimisation. Conversely, engineering models, typically based on one-dimensional beam structural models coupled with BEM-based aero- or hydrodynamic solvers (e.g. OpenFAST (Jonkman, 2013), HAWC2 (Larsen and Hansen, 2007)), are preferred in engineering practice due to their relatively low computational cost. Such models necessarily involve simplifying assumptions that may not capture all of the important FSI physics of the problem being modelled. In particular, our previous work highlighted that the simplifications involved in engineering models based on blade-element theory can induce significant errors when flapwise deformations are observed (Zilic de Arcos et al., 2022).

Both flapwise and twist deformation components can produce significant changes in the hydrodynamics of a tidal turbine blade (Zilic de Arcos et al., 2022). Twist deformations change the spanwise distribution of angle of attack, and thus forces along the blade. Even modest twist deformations can create a significant impact on blade thrust and power, effects that can largely be described using blade-element theory. On the contrary, flapwise deformations, in absence of twist deformations, generate two different and opposing hydrodynamic effects on the blade. Firstly, the bent blade creates a radial force on the flow which increases the radial velocity and wake expansion, thereby generating a reduction in static pressure on the suction surface and increasing loads on the inboard sections of the blade. Secondly, due to the increased spanwise flow along the blade, the onset of near-tip losses move inboard, increasing the load shedding on sections closer to the blade tip. These are effects of a three-dimensional nature that were shown to modify shape of the lift and drag curves, as functions of angle of attack, along the span of the blade, with a consequential impact on power and thrust. In particular, for the analysed tidal rotor and range of flapwise deformations at the tip (from 7.5% to 17.5% of blade radius), we observed modest variations in integrated thrust coefficients (−4% to ＋2%) but a substantial reduction in integrated power performance of up to a 20% for bent blades with identical twist distributions (Zilic de Arcos et al., 2022).

This paper builds on the hydrodynamic mechanisms of flawise deformations, described in our previous work (Zilic de Arcos et al., 2022), to present a study on the hydrodynamic interactions between the flapwise and twist deformation components on a tidal rotor. The study is based on a set of parametrically-deformed steady-state CFD simulations of a tidal rotor blade. The simulations are analysed to determine whether the two deformation components can be regarded as hydrodynamically independent of each other, and to determine the implications of the hydrodynamic changes associated with blade deformations for lower-fidelity models, which is done through the re-examination of some fundamental constituents of such models.

The discussion is divided in two sections: Section 3 discusses the fundamental fluid dynamics, independence and implications of coupling the two deformation components, noting possibilities for the development and improvement of BEM-based engineering models. This is followed, in Section 4, by a practical application of our findings, where flapwise and twist deformations are used to determine possible passive control strategies for a 20m diameter tidal rotor, highlighting the importance of adequately capturing the effects of both twist and flapwise deformations.

## 2 Methods

### 2.1 Rotor characteristics

A 20m diameter tidal rotor designed for a blocked-flow condition was employed in this analysis. The blades were designed by Schluntz and Willden (2015) for an optimum tip-speed ratio λ=5.5, where $\lambda =\omega R/V_{\infty }$, with ω the rotational speed, R the rotor radius and $V_{\infty }$ the undisturbed flow speed, operating under an isotropic blockage ratio (defined as the ratio between the turbine swept area and the channel cross sectional area) of B=0.196.

**Fig. 2:**
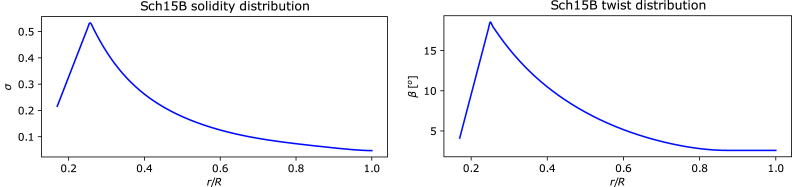
Characteristics of the Sch15B rotor: Spanwise distributions of the blade solidity σ (left), and twist angle β (right).

The blade geometry consists of a single Risø A1-24 aerofoil section centred around the quarter-chord line from 25% of the span to the tip. The spanwise distribution of blade solidity (σ(r)=Nc(r)/2πr, with N the number of blades, c(r) the local chord and r the local radius) and twist angles β(r) are presented in Fig. 2. Further details about the rotor geometry and design methodology can be found in Schluntz and Willden (2015).

### 2.2 Blade deformation parameter space

The rotor blade geometry was parametrically deformed to obtain an array of deformation cases accounting for flapwise-only, twist-only and flapwise–twist coupled deformation cases, in addition to the original undeformed rotor blade. The geometries were generated using a CAD model with the algorithmic-aided design software Rhinoceros 6.0 with Grasshopper 1.01.

The deformed shapes were obtained by scaling the static response of a shell-based structural model under realistic loading conditions, described by Zilic de Arcos et al. (2018), for the flapwise deformations, and through the prescription of a linear distribution of twist deformations along the span that modify the original twist distribution. For the flapwise deformation, the shape of the deformed quarter-chord line was scaled to achieve a desired deformation at the tip. The twist deflections are prescribed linearly from r/R=0.25 towards the tip, where the maximum deformation is encountered. Using a linear twist deformation distribution is a simplification of a complex fluid-structural phenomenon that involves the moment distribution over the blade, the position of the structural centre and the bending–twist coupling mechanisms (Lobitz and Veers, 2003). Each analysed case was labelled based on the flapwise and twist deformations at the tip, δx/R and δβ, respectively.

**Fig. 3:**
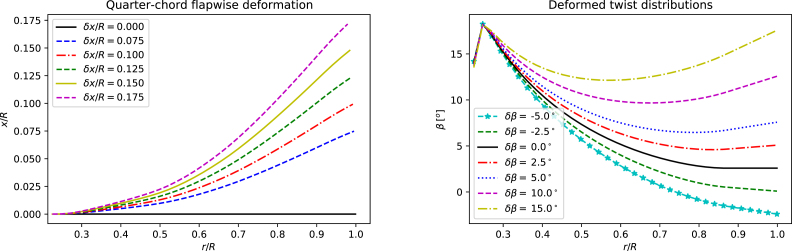
Spanwise distribution of the axial-position of the quarter-chord line for different flapwise deformation cases (left) and spanwise twist distribution for different twist deformation cases (right).

The blade surfaces were generated for six twist-only cases (tip twist angles δβ between −5.0° and +15.0°, with positive values deforming towards feather), 5 flapwise-only cases (tip deflection δx/R between 0.075 and 0.175), and 5 cases with coupled deformations, in addition to the original rotor geometry referred to as the undeformed case. The flapwise deformations and the deformed twist distributions over the span are presented in Fig. 3. Each deformation case was simulated at four tip-speed ratios (λ∈{4.0,5.0,6.0,7.0}). A summary of simulated cases is presented in Table 1.

**Table 1:** Testing matrix of simulated cases for twist (δβ) and flapwise (δx/R) deformations. Each point in this matrix was simulated the tip-speed ratios λ∈[4.0,5.0,6.0,7.0]. The point highlighted in red corresponds to the undeformed case.

δx/R	δβ[°]						
	−5.0	−2.5	0.0	2.5	5.0	10.0	15.0
0.000	✓	✓		✓	✓	✓	✓
0.075		✓	✓	✓			
0.100			✓	✓	✓		
0.125			✓				
0.150			✓				✓
0.175			✓				

### 2.3 CFD modelling

The simulation strategy presented in this work follows the work presented in Zilic de Arcos et al. (2022). The simulations were performed using a RANS (Reynolds-Averaged Navier Stokes) CFD (Computational Fluids Dynamics) model with a k−ω SST turbulence model (Menter, 1994, Menter et al., 2003) under steady-state assumptions and using the Luo and Gosman (1994) MRF (Multiple Reference Frame) model. The model discretisation consists of a cell-centred finite volume scheme with second-order spatial discretisation and a coupled velocity–pressure algorithm for the solution of the equations. The computations were made with the commercial software ANSYS Fluent 19.0.

A 120° segment of a cylindrical domain with a diameter equivalent to 10 times the rotor diameter was used, ensuring low blockage effects (blockage of 1%), and with boundary conditions defined as: velocity inlet (4.5m/s, turbulence intensity of 10% and length scale 0.7 times the rotor diameter), pressure outlet, symmetry condition for the outer domain walls, periodic conditions that limit the effective solution of the equations to a 120° wedge, and non-conformal interfaces between the two subdomains, as described in Zilic de Arcos et al. (2022). This is a widely-used simulation strategy previously validated by Wimshurst and Willden (2017) in terms of integrated thrust and power coefficients, spanwise force distributions, and sectional pressure coefficients.

Structured meshes were built for every case using ICEM 19.0, preserving the grid topology around the blade surfaces between cases and adapting buffer regions up- and downstream of the rotor to allow blade deformations. The number of cells between the different deformation cases was kept constant. A base mesh for the rotor domain, corresponding to an intermediate mesh resolution, was prepared. The blade surface was discretised with 128 cells over the chord, 110 cells in the spanwise direction, and the non-dimensional wall distance $y^{+}$ was kept within the wall-modelling region (i.e., $30\leq y^{+}\leq 300$). The near-wall O-grid region had a first-layer thickness close to the blade surfaces of $\Delta y=2.5\times 10^{-4}$m (giving $8.5\times 10^{-5}\leq \Delta y/c\leq 2.5\times 10^{-4}$ along the blade), and a wall normal growth-rate of 1.05 over 25 layers that smoothly transitions to the rest of the domain.

The mesh sensitivity was evaluated using the Grid Convergence Index (GCI) introduced by Roache (1994). To quantify the discretisation error, this method compares the values of a quantity of interest (in this case, power and thrust coefficients) to the extrapolated value where the number of elements tends to infinity. Two meshes, a coarser and a finer one, were generated from the baseline (intermediate) mesh for the undeformed rotor geometry. The grids were prepared by applying a homogeneous refinement factor $k_{i}$ on each dimension, leading to 0.70, 5.41, and 43.28 million cells for the coarse, intermediate and fine meshes, respectively. Table 2 presents a summary of this study, where the GCI calculated for the power and thrust coefficients ($C_{P}$ and $C_{T}$, respectively, defined in Section 2.4) using a safety factor $F_{S}=2$. From these results, the intermediate mesh resolution is deemed appropriate due to its low discretisation uncertainty and reasonable computational costs. The full extent of the mesh sensitivity study is described further in Zilic de Arcos (2021).

**Table 2:** Mesh sensitivity analysis for the turbine domain, where $n_{c}$ is the number of elements, $k_{i}$ the refinement factor, $E_{R}$ the relative error between two meshes, and CGI the grid convergence index for the thrust and power coefficients $C_{T}$ and $C_{P}$, respectively.

# of elements	Refinement factor	Thrust			Power		
$n_{c}$	$k_{i}$	$C_{T}$	$E_{R}$	GCI	$C_{P}$	$E_{R}$	$GCI_{i}$
		[–]	[%]	[%]	[–]	[%]	[%]
0.70M	1.00	1.024	–	–	0.297	–	–
5.41M	2.00	1.064	3.72	1.54	0.345	13.95	0.99
43.28M	4.00	1.059	−0.50	0.54	0.344	−0.32	0.35

∞	∞	1.062	0.00	0.00	0.344	0.00	0.00

### 2.4 Simulation post-processing

The spanwise force distributions were calculated integrating pressure (p) and shear stresses ($\vec{S}$) over the aerofoil sections at different radial locations. The resulting forces are then presented as components in the global frame of reference:

(1) $$\vec{F}\left(r\right)=\oint \left(p\overset{ˆ}{n}+\vec{S}\right)dC$$

with the integral solved over a blade slice C at r, where p is the static pressure, and $\overset{ˆ}{n}$ the blade surface normal vector.

The power and thrust coefficients were calculated, respectively, as:

(2) $$C_{P}=\frac{P}{1/2\rho AV_{\infty }^{3}}=\frac{\omega \int F_{\theta }\left(r\right)rdr}{1/2\rho AV_{\infty }^{3}}$$

(3) $$C_{T}=\frac{T}{1/2\rho AV_{\infty }^{2}}=\frac{\int F_{x}\left(r\right)dr}{1/2\rho AV_{\infty }^{2}}$$

where P and T are the integrated power and thrust, $F_{x}$ and $F_{\theta }$ are components of $\vec{F}\left(r\right)$ in the axial and tangential directions respectively, ρ the fluid density, A the undeformed turbine swept area, and $V_{\infty }$ the undisturbed flow velocity.

Flow quantities, including induction factors and angles of attack, were sampled from the simulated flow using the Streamtube Averaging Method (SAM). This is a methodology that allows the determination of flow variables at the rotor plane based on an interpolation over a streamtube using the flow data up- and downstream from a rotor section, avoiding the strong gradients that arise near the rotor blade itself. The method accounts for the expansion of the wake to sample the flow, as described by Zilic de Arcos et al. (2020b).

The data used for the interpolation was extracted at an axial distance of x/c=±1.0 from the blade . The local deformation was accounted for, both to determine the location of the intersection between the streamtube and the rotor, as well as to establish the axial coordinates for interpolation.

The inflow angle ϕ and the local angle of attack α, for a blade section at r/R, were calculated at each radial location using the axial and tangential velocities sampled from the simulated flow, $V_{x}$ and $V_{\theta }$. The inflow angle ϕ and the angle of attack α were determined as:

(4) $$\phi \left(r\right)=\tan^{-1}\left(\frac{V_{x}\left(r\right)}{V_{\theta }\left(r\right)}\right),$$

and

**Fig. 4:**
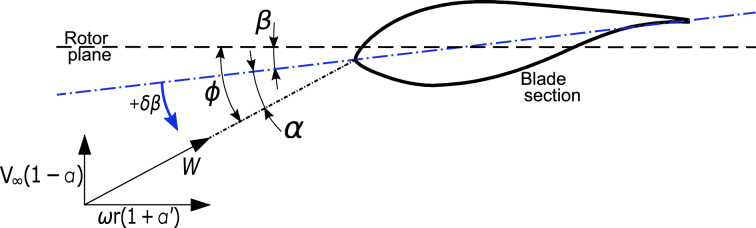
Flow diagram over an arbitrary section of the blade, as described by BEM theory.

(5) α(r)=ϕ(r)−β(r)

as displayed in Fig. 4.

The interference of the rotor in the streamwise flow is represented by the axial induction factor a, defined as:

(6) $$a\left(r\right)=1-\frac{V_{x}\left(r\right)}{V_{\infty }}$$

Finally, the flow velocities, angles of attack and inflow angles were used, in conjunction with the spanwise distribution of thrust and tangential forces, to determine the local lift and drag coefficients.

## 3 Hydrodynamic independence of twist and flapwise deformations

Blade element theory is a framework which, by assuming that the forces on a three-dimensional blade can be explained as a collection of locally two-dimensional foils, allows prediction of the performance and load changes on a blade undergoing twist changes, either in the form of twist deformation or blade pitching. Many engineering tools, as discussed in Section 1, rely on this assumption to predict blade loads and performance.

Our previous research, however, demonstrated that the effects of flapwise deformation on thrust and torque can be significant, with causes that cannot be attributed simply to changes in angles of attack, relative inflow velocity or vector re-orientations (Zilic de Arcos et al., 2022). This suggests that models based on two-dimensional blade element theory cannot capture the hydrodynamic effects of flapwise deformations, nor can these effects be exploited by design methods based on blade element theory.

In the following sections we study the hydrodynamics of flapwise and twist deformations by looking at the effects of twist-only, flapwise-only and coupled flapwise and twist deformations on blade hydrodynamics. The focus of the analysis is to explore the independence and couplings between flapwise and twist deformation hydrodynamics, and the implications of such couplings for BEM-based models.

### 3.1 Force analysis

**Fig. 5:**
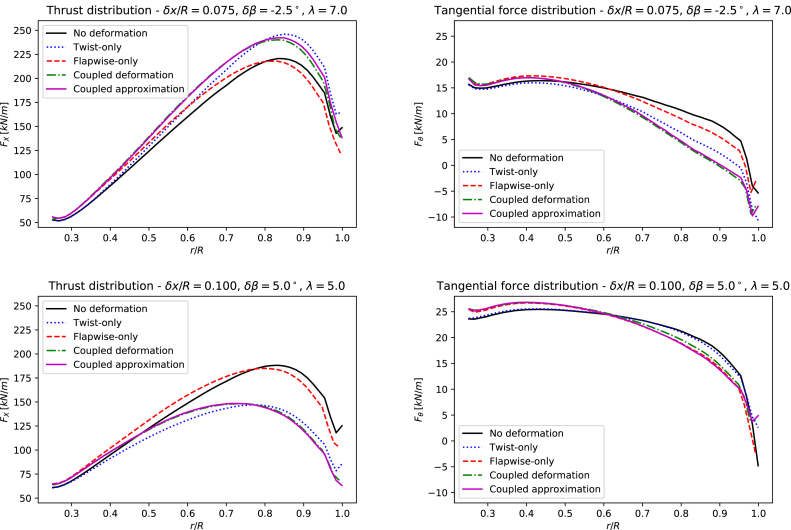
Evaluation of the hydrodynamic independence hypothesis in terms of thrust (left column) and tangential force per unit span (right column) for two different sets of blade deformations, at different tip-speed ratios (top row: δx/R=0.075,δβ=−2.5°, λ=7.0; bottom row: δx/R=0.100,δβ=5.0°, λ=5.0.). (For interpretation of the references to colour in this figure legend, the reader is referred to the web version of this article.)

Fig. 5 shows the spanwise thrust and tangential force distributions per unit span on a rotor blade for two sets of twist-only, flapwise-only, no deformation and coupled flapwise and twist deformation cases.

The twist-only and flapwise-only cases in both sets show the trends described in previous research (Zilic de Arcos et al., 2022): the twist-only cases show changes on the spanwise force distribution due to local changes in angle of attack, seen as a monotonic increase (δβ=−2.5°) or decrease (δβ=+5.0°) in thrust, alongside reductions in tangential force along the span. The flapwise-only cases show a load augmentation at the inboard sections and an increase in load shedding near the tip.

The thrust and tangential forces in the coupled deformation cases appear to be dominated, in most cases, by the twist deformation effects. However, comparing the twist-only and coupled deformation cases also reveals the inboard load augmentation and near-tip load shedding effects that are characteristic of the flapwise deformation.

These observations are confirmed by isolating the effects of the flapwise and twist deformations, which are calculated as the difference in local thrust and tangential force between undeformed and deformed cases. Assuming the deformation effects to be independent from each other leads to the spanwise force distributions of a blade with coupled flapwise and twist deformations to be estimated as:

(7) $$F_{CD}^{*}=F_{ND}+\left(F_{TO}-F_{ND}\right)+\left(F_{FO}-F_{ND}\right)$$

where $F_{CD}^{*}$ is an approximation of the spanwise force distribution of a case with coupled deformations, for either thrust or tangential force. The sub-indices CD, ND, TO and FO stand for coupled deformation, no deformation, twist-only and flapwise-only, respectively.

The results of Eq. (7) are shown in Fig. 5 under the label of ‘Coupled approximation’. The plots show good agreement between the simulated case with coupled deformations and the approximation $F_{CD}^{*}$ for both tangential force and thrust.

**Fig. 6:**
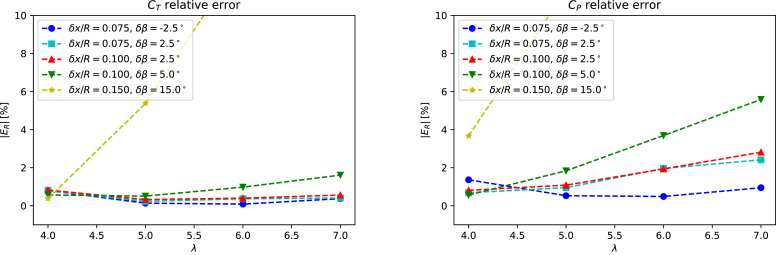
Relative error of the integrated power and thrust coefficients between the coupled deformation CFD results and an approximation based on the twist-only and flapwise-only results (Eq. (7)).

A further analysis of the accuracy of Eq. (7) can be seen in Fig. 6. These plots show the relative error, calculated in terms of $C_{P}$ and $C_{T}$, for the different cases simulated with coupled deformations at varying tip-speed ratios. The relative error plots confirm that assuming the force changes produced by the flapwise-only and twist-only deformations to be independent from each other produces good agreement with the CFD simulations for moderate blade deflections. The results show a maximum relative error of 5.5% on $C_{P}$ and below 2.0% on $C_{T}$ for all cases except where {δx/R=0.150,δβ=15°}. This case, the largest deformation simulated, shows a relative error in excess of 10%, suggesting significant interactions and higher-order effects. Nevertheless, for moderate blade deflections, the relatively low error observed suggests that the hydrodynamic effects of flapwise and twist deformation can be regarded as independent of each other.

In the following sections, we analyse the impact of this hypothesis in terms that are relevant for the development and improvement of engineering models, especially those based on BEM theory.

### 3.2 Axial induction factors

The relationship between the local thrust coefficient and the axial induction factor a is an important component of BEM models. This is typically represented by the axial momentum equation below a critical value $a_{c}$, and by an empirical function above. The empirical model is used to account for turbulent wake effects in high-load regimes where the validity of the analytical model breaks down (Burton et al., 2011).

**Fig. 7:**
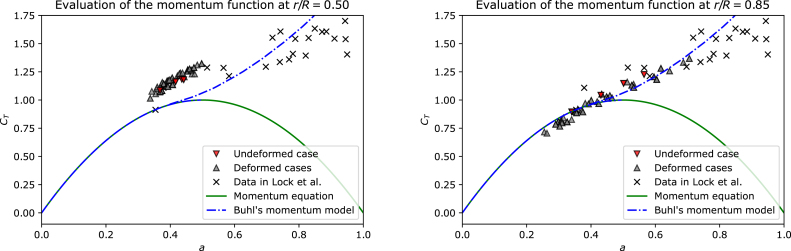
Relationship between the sectional thrust coefficients and the axial induction factors for different cases around the inboard (r/R=0.50R, left) and approaching the tip of the blade (r/R=0.85, right). Buhl’s momentum model (dot-dash line) and the analytical axial momentum model (solid line) are plotted for reference.

Fig. 7 presents the relationship between axial induction factor and local thrust coefficient. The data points extracted from the CFD simulations, for different deformation cases, are displayed at two radial locations. Alongside the CFD data, the figure also shows the BEM analytical thrust model (Burton et al., 2011), the widespread empirical model for turbulent wake state of Buhl (2005) (used by, e.g. Ning (2014) and Jonkman et al. (2015)), and the experimental data of Lock et al. (1926) reproduced from Buhl (2005).

The range spanned by the CFD data in Fig. 7 is a result of the parameter space described by the different cases simulated (λ, δx/R, δβ). This spans a smaller range of a compared to the experimental data from Lock et al. (1926), and shows that blade deformations do not appear to affect significantly the functional relationship between thrust and axial induction factors in this range. However, the CFD data also highlights that radial location has a more significant impact on this function than blade deformations.

The relatively small spread in the CFD data with and without deformations, compared with the data in Lock et al. (1926), suggests that the thrust function used for an undeformed blade should be sufficient to model a rotor with blade deformations within a BEM framework. The momentum model derived by Buhl (2005), however, differs considerably from the CFD results especially around the inboard sections, which may be a potential source of error in BEM computations. The development of a correction to this model is beyond the scope of the this paper and will be addressed in future work.

### 3.3 Angles of attack

**Fig. 8:**
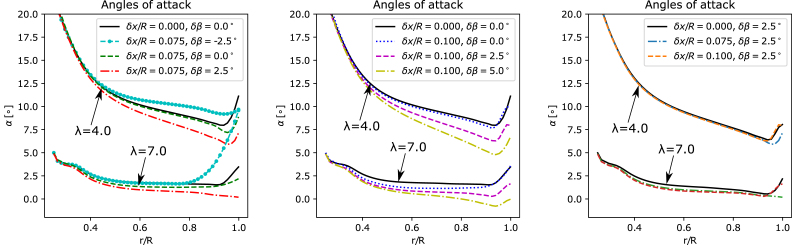
Spanwise distribution of angles of attack for three sets of deformation cases, each presented at two different tip-speed ratios. From left to right: varying δβ with δx/R=0.075; varying δβ with δx/R=0.100; and varying δx/R with δβ=2.5°. The three sets are compared with the undeformed case (solid black line). (For interpretation of the references to colour in this figure legend, the reader is referred to the web version of this article.)

Fig. 8 shows the spanwise distribution of angles of attack for three sets of deformation cases at tip-speed ratios of 4.0 and 7.0. The first two sets include cases with varying twist deformations and fixed δx/R, alongside the undeformed case. The third set presents a fixed twist deformation at different δx/R cases.

Twist deformations affect the angle of attack by altering the relative angle of the blade sections to the incoming flow. The change in α is different to that of the twist deflection due to the adjustment of the inflow angle. Positive (negative) twist deformations act to reduce (increase) angle of attack, typically reducing (increasing) thrust force and thus increasing (reducing) the axial flow speed and therefore the inflow angle ϕ.

The results demonstrate that flapwise deformations have a limited effect on the α distribution despite the force changes on both the inboard and near-tip sections of the blade. The inboard α variations are more evident for the high tip-speed ratio cases, as shown in Fig. 8 (right). Small reductions in the inboard angle of attack are observed as δx/R increases due to the reduction in axial flow speed given the inboard load augmentation. As the blade tip is approached, the difference between the cases with and without flapwise deformation decrease, since ϕ and thus α are increasingly dominated by the rotational speed. Near the tip (r/R≥0.90) divergence between the cases is observed. This is an area of the blade where the concept of a two-dimensional angle of attack, in a flow dominated by three-dimensional flow effects, is no longer applicable (Wimshurst and Willden, 2018).

### 3.4 Polar coefficients

**Fig. 9:**
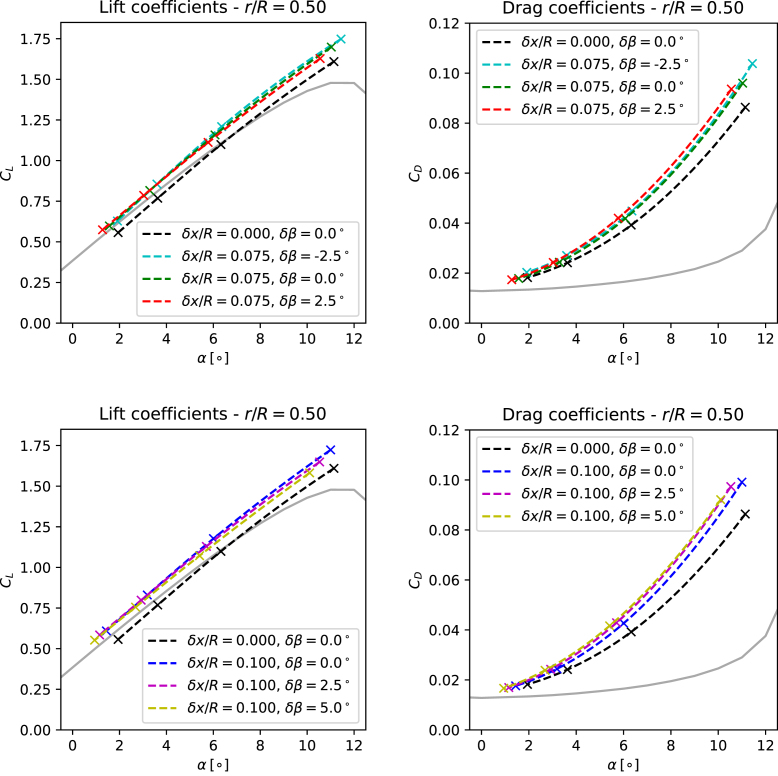
Polar lift (left) and drag (right) coefficients extracted from blade-resolved simulations extracted at r/R=0.50 for different deformation cases. Two-dimensional polars plotted for reference (grey solid line). (For interpretation of the references to colour in this figure legend, the reader is referred to the web version of this article.)

**Fig. 10:**
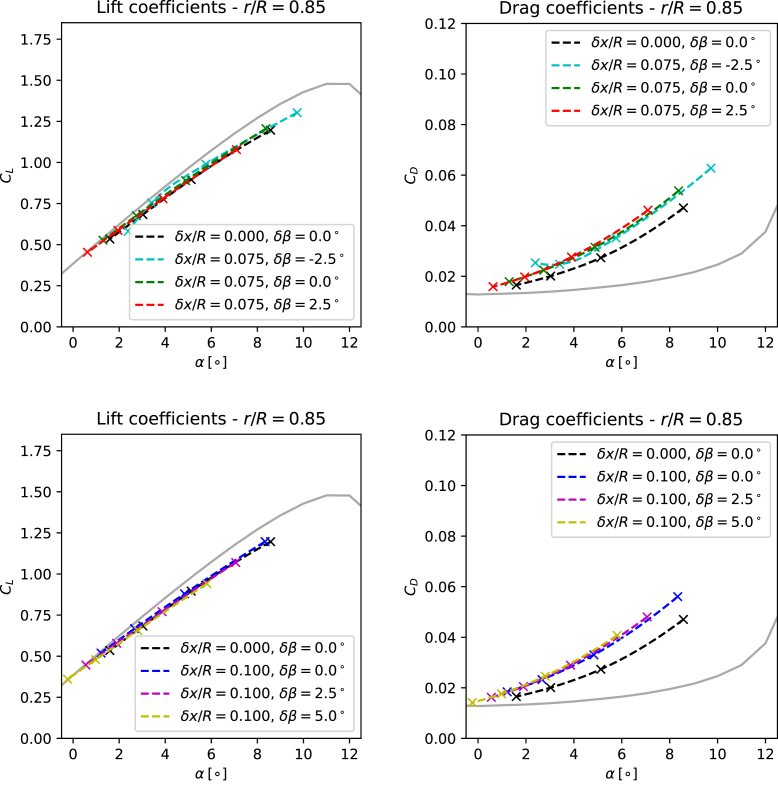
Polar lift (left) and drag (right) coefficients extracted from blade-resolved simulations close to the tip of the blade (r/R=0.85) for different deformation cases. Two-dimensional polars plotted for reference (grey solid line). (For interpretation of the references to colour in this figure legend, the reader is referred to the web version of this article.)

Fig. 9, Fig. 10 show lift and drag coefficients for the same sets of deformations described above. The coefficients were extracted from the blade-resolved CFD results and are presented for two spanwise locations: r/R=0.50 and r/R=0.85. Each data point corresponds to a specific λ, with $C_{L}$ and $C_{D}$ reconstructed considering the local flow and blade characteristics as described in Section 2.4. Interpolating splines were added to the plots to reflect the general $C_{L}$ and $C_{D}$ dependency upon the angle of attack α.

Fig. 9 shows at r/R=0.50 the inboard load augmentation caused by flapwise deformation for δx/R=0.075 and δx/R=0.100. This is observed as a significant step-increase in the lift coefficient, and an increase in drag dependent on α and δx/R. The influence of twist deformation in lift and drag is much smaller, especially at lower angles of attack, and is considered negligible.

Closer to the tip, the lift curves converge with each other for cases with and without flapwise deformation . This suggests that the inboard load augmentation previously mentioned, is counteracted by the onset of the tip-loss effects moving inboard. The cases with flapwise deformation still present an attendant increase in drag in the near-tip region. This effect, showing a small dependency on twist, can also be attributed to the tip-loss effects moving inboard, and the associated drag increase described by Wimshurst and Willden (2018) due to tip-loss mechanisms influencing the outboard sections of the blade.

### 3.5 Hydrodynamic centre of pressure

The position of the hydrodynamic centre of pressure is relevant for the design and implementation of passive control strategies. These strategies rely on achieving desired deformations, using a bespoke or tailored structure, to fulfil specific control objectives. From an engineering perspective, if the blade loads and deformations are known, tailoring a structure involves solving an inverse structural problem where the structural and hydrodynamic centres are important variables. The centre of pressure is the location on the blade section around which the hydrodynamic forces are centred, and the structural centre depends on structural design and material selection. The offset between these two points along the blade creates a torque through the span which induces twist deformations.

**Fig. 11:**
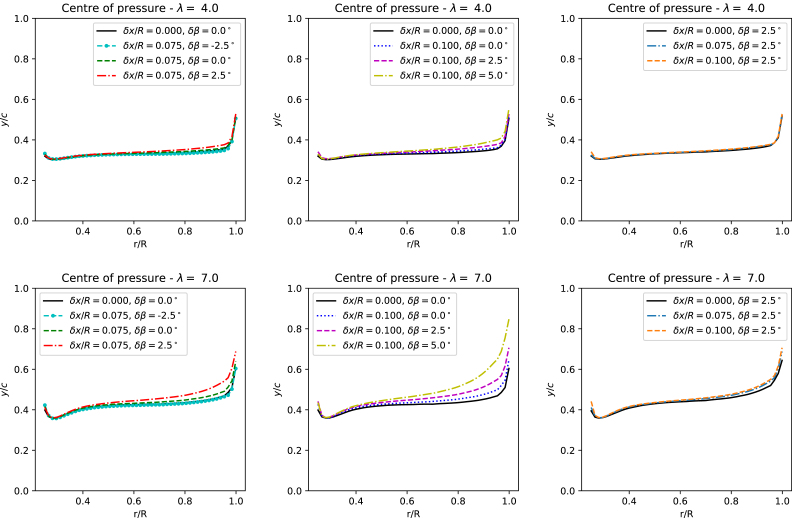
Spanwise distribution of the hydrodynamic centre of pressure for three sets of deformation cases at λ=4.0 (top row) and λ=7.0 (bottom row). The centre of pressure is plotted over a local tangential coordinate y, measured from the leading edge, and non-dimensionalised by the local chord. From left to right: varying δβ and δx/R=0.075; varying δβ and δx/R=0.100; and varying δx/R with δβ=2.5°.

As with the angles of attack, the position of the hydrodynamic centre appears to be dominated by twist rather than flapwise deformation. Fig. 11 shows that twist deflections acting to reduce the angle of attack (i.e. positive δβ) shift the centre of pressure towards the trailing edge and vice-versa, with larger deformations driving the largest shift in the centre position. The results also show an influence of tip-speed ratio, as the centre shifts are larger for the cases operating at λ=7.0 than for λ=4.0.

Analysing the set of cases with fixed δβ and varying δx/R, we observe that flapwise deformations have a limited effect on the centre of pressure, with a maximum relative change of ca. 5% near the tip shifting towards the trailing edge, despite the significant changes in the polar coefficients discussed in Section 3.4, and the implied changes to pressure distribution along the chord.

### 3.6 Summary

The analysed results show that, for moderate blade deflections, the hydrodynamic effects induced by the flapwise and twist deformation components can be approximated to be independent from each other. In terms of the constituents of BEM theory, the relationship between thrust and axial induction factor shows no significant influence from blade deformations, while two-dimensional polar coefficients are affected only by flapwise deformations. These changes in lift and drag, which affect the functional relationship of $C_{L}$ and $C_{D}$ with α, show no significant coupling effects between flapwise and twist, nor a significant influence of twist deformations, for moderate blade deformations.

Angles of attack and centres of pressure showed negligible interactions between twist and flapwise deformation, with changes dominated by twist deformations. Flapwise deformations showed a very limited influence in these quantities.

The hydrodynamic independence of the two deformation components allows the estimation of the forces on a rotor blade with coupled flapwise and twist deformations using twist-only and flapwise-only data, with a relatively low error for moderate deflections. The implications of this will be demonstrated in the following sections, where we explore passive control strategies for a tidal rotor.

## 4 Design of passive load alleviation control strategies

Passive blade deformation strategies can be used to alleviate both thrust and torque loads on rotor blades, which need to be designed specifically for this purpose. Designing a passively deforming blade for load alleviation requires that an inverse structural problem be solved where the deformations and loads are known *a priori* to tailor a suitable structure. Our approach to this problem consists in focusing on the hydrodynamics effects of blade deformations to find a preliminary passive control path for the analysed rotor defined by a set of $\left(C_{P},C_{T}\right)=f\left(\delta x/R,\delta \beta ,\lambda \right)$ points, where each point corresponds to a flow speed between the cut-in and cut-out speeds.

The parameter space $\left(C_{P},C_{T}\right)=f\left(\delta x/R,\delta \beta ,\lambda \right)$ was defined by combining the available CFD data (which includes the full range of twist-only and flapwise-only cases alongside a selection of cases with coupled twist and flapwise deformations, as described in Section 2.2), with the results of Eq. (7) used to estimate the blade loads of the coupled deformation cases that were not simulated. Integrating the forces leads to a fully-defined parameter space for $C_{P}$ and $C_{T}$ in terms of δx/R, δβ and λ, where control paths can be designed.

To find suitable control paths we specified target thrust curves for each strategy that monotonically increase with flow speed. These target thrust curves are considered to be the key input for two passive control strategies, since thrust is the main driver for blade deformations. The control paths are designed for a rotor expected to operate in currents between 0.5 to 4.5m/s, and with a rated flow speed of 3.0m/s. Note that this thrust-based approach contrasts the typical focus on power in the design of active-pitch control strategies.

The target thrust curves are defined by a fixed thrust coefficient $C_{TR}$ below the rated flow speed and a linear function between $V_{R}$ and the cut-out speed $V_{CO}$, which is expected to correspond to the maximum thrust $T_{max}$. The thrust curves are thus fully determined by the cut-in, rated and cut-out flow speeds ($V_{CI}$, $V_{R}$ and $V_{CO}$, respectively), the thrust coefficient at rated flow speed, and the maximum thrust.

**Table 3:** Comparison between the operational target parameters of three different control strategies.

Strategy	$C_{TR}$	$T_{max}$	$\delta x_{max}/R$	$V_{R}$
	[–]	[kN]	[–]	[m/s]
*Passive control 1*	0.70	1,450.00	0.10	3.00
*Passive control 2*	0.80	1,600.00	0.15	3.00
*Active pitch control*	0.91	1,282.00	0.00	3.00

Two thrust curves are considered for two different passive control strategies to demonstrate this approach. These two curves allow exploration of the trade-off between $C_{T}$ and $C_{P}$, with higher thrust values typically enabling higher power coefficients but leading to higher maximum loads. The design variables defining the two curves are shown in Table 3 in addition to the maximum allowable flapwise deformation $\delta x_{max}/R$. Alongside the thrust parameters for the passive strategies, reference values for an active pitch controlled turbine rated at 2MW are provided.

**Fig. 12:**
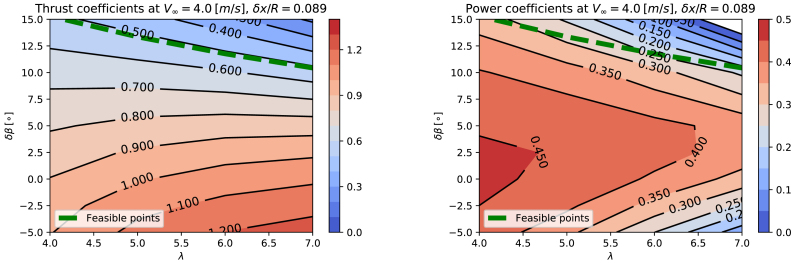
Contour plots of thrust (left) and power (right) coefficients for the Passive control 1 case at an inflow speed of $V_{\infty }=4.0\text{m/s}$, with $V_{\infty }\geq V_{R}$, and under a flapwise tip deflection of δx/R=0.089. Highlighted in green, the locus of feasible points where the desired thrust is achieved. (For interpretation of the references to colour in this figure legend, the reader is referred to the web version of this article.)

The flapwise deformation is assumed to be proportional to thrust ($\delta x\propto V_{\infty }^{2}$), with the maximum allowable thrust and flapwise deformation defined as design variables. This allows the three-dimensional parameter space {δx/R,δβ,λ} to be reduced to a two-dimensional space where $C_{T}$ and $C_{P}$ are functions of δβ and λ. At a flow speed $V_{i}$, the $C_{T}$ required to meet the target thrust profile is determined and the corresponding set of δβ,λ that achieve the required $C_{T}$ can be identified. The set of {δβ,λ} where the target thrust is fulfilled is defined as the locus of feasible points for the flow speed $V_{i}$. Fig. 12 shows a typical example of the thrust and power planes obtained after reducing the parameter space for a flow speed above rated $\left(V_{\infty }=4.0\text{m/s}\right)$. This figure also highlights the locus of feasible points on both contours. Specifically, the locus of feasible points on the power contour shows how $C_{P}$ varies for a constant value of $C_{T}$. A $C_{P}$ and corresponding {δβ,λ} pair is selected amongst the locus of feasible points that best matches the $C_{P}$ objective at a given flow speed (power maximisation below $P_{R}$ or power shedding to limit power capture in excess of $P_{R}$). Consequently, the required deformed blade geometry (δβ and estimated δx/R) and rotational speed λ that satisfy the required thrust, alongside with the best possible match to the desired $C_{P}$ within the operating constraints, are defined as functions of each flow speed $V_{i}$ between cut-in and cut-out.

### 4.1 Passive hydrodynamic load alleviation

**Fig. 13:**
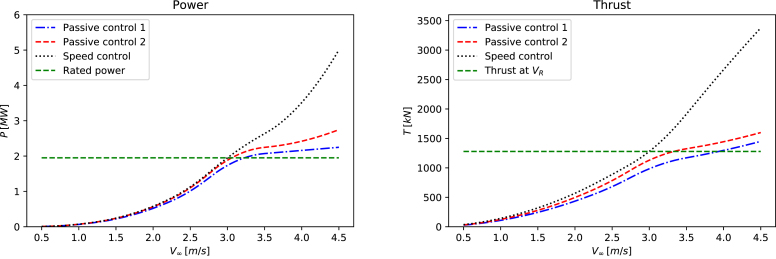
Power and thrust as functions of the freestream flow speed $V_{\infty }$. The plots show the two passive control strategies, a rigid blade with speed control, and reference values that correspond to a rigid rotor with active pitch control. The rated flow speed $V_{R}=3.0\text{m/s}$.

Fig. 13 shows power and thrust as functions of the flow speed for the passive control strategies, designed with the methodology described above and defined by the parameters presented in Table 3. Alongside the passive control strategies, two sets of results corresponding to the original blade design, assumed to be rigid, are provided for comparison: a speed-controlled rotor operating within the limits of the parameter space (4.0≤λ≤7.0); and reference values for rated power and thrust at rated flow speed, values taken to characterise an active-pitch controlled rotor.

The results shown in Fig. 13 highlight that passive control paths were found to fulfil the targeted thrust constraints across all flow speeds. The lower thrust values associated with the passive strategies, compared with the rigid speed-controlled rotor, result in a slightly lower power below rated flow speed. The advantage of the passive control is realised above the rated flow speed, where significant load shedding and power capping are achieved by both passive strategies when compared with the speed-only control of the rigid rotor.

The power at cut-out speed ($V_{CO}=4.5\text{m/s}$) for the speed control case exceeds $P_{R}$ by 150%. 55% and 45% of this power is shed by the passive control strategies 1 and 2, which exceed $P_{R}$ at $V_{CO}$ by only 12% and 37%, respectively. Note that the difference in maximum power between the two passive strategies is a consequence of the higher maximum thrust of the passive strategy 2 defined from design.

**Fig. 14:**
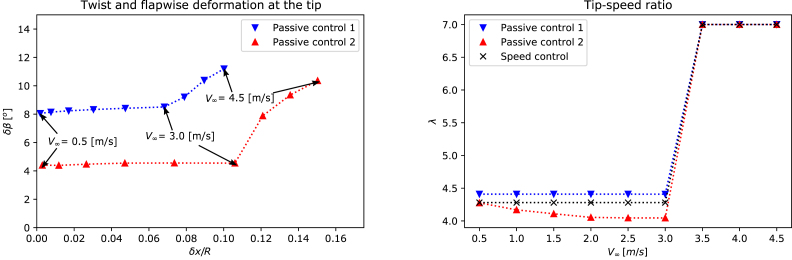
Blade twist deformation δβ as a function of blade tip deformation δx/R for the two passive control cases (left) and tip-speed ratios as functions of the incoming flow speed required to provide passive turbine control. The required deformations are indicated at 0.5m/s increments between the cut-in $V_{CI}=0.5\text{m/s}$ and cut-out $V_{CO}=4.5\text{m/s}$ flow speeds.

The blade deformation and control paths as functions of flow speed for the two passive control strategies, followed to achieve the power and thrust curves, are shown in Fig. 14. This shows that a significant twist deformation (relative to the rigid blade geometry) is required at cut-in speed $V_{CI}$ to ensure the low $C_{TR}$ required by the strategy design. In practice, this implies that the rotor needs to be pre-twisted, altering the original spanwise twist distribution originally optimised for power capture by a rigid blade. The pre-twist in this work is considered as a spanwise-varying modification of the original twist distribution, equivalent to the deformations described in Section 2.2, that leads to rotor blades that are different from the original design when no forces are applied.

The required pre-twist was found by extrapolating the deformation curves to $V_{\infty }=0.00\text{m/s}$. The resulting blade tip pre-twists are $\delta \beta {|}_{V_{\infty }=0\text{m/s}}=7.99{}^{\circ}$ for the passive control strategy 1 and $\delta \beta {|}_{V_{\infty }=0\text{m/s}}=4.41{}^{\circ}$ for the passive control strategy 2.

The deformations shown in Fig. 14 demonstrate that the objectives achieved with the passive control strategies in terms of thrust shedding and power capping can be realised with relatively modest changes in blade twist up to the cut-out velocity. This range is approximately 3.5° for the passive control strategy 1 and 6.0° for the passive control strategy 2. These maximum twist deformations lie in a similar range to those considered feasible in the literature reviewed in Section Section 1 (approximately 2.5° to 5.0°).

Fig. 14 also shows the tip-speed ratio as function of $V_{\infty }$ for the two passive control strategies and the speed control case. The three cases show the same trend: below rated, the tip-speed ratio is maintained low and at its local optimum; while above $V_{R}$ the control approach quickly saturates at the maximum possible λ to shed power. As λ reaches its maximum limit in the three cases, the power and load shedding is achieved through blade deformations. These deformations lead to the passive control strategies outperforming the speed control case in terms of limiting power absorption and load shedding.

### 4.2 Contribution of deformation components to passive control

**Fig. 15:**
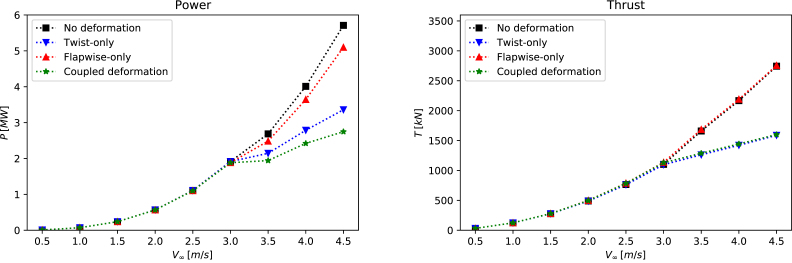
Contribution of blade deformation components to generated power (left) and thrust (right). The results presented correspond to the passive control 2 strategy.

Fig. 15 shows the power and thrust as a function of flow speed corresponding to the passive control 2 case, which has the largest flapwise deformations, separated by the different deformation components. The points were calculated for the deformations and tip-speed ratios from the passive control path at the corresponding flow-speeds. The pre-twist was included for all deformation components.

The results show that power and thrust shedding is dominated by the twist deformation above rated flow speed. Although flapwise deformation has a negligible impact on thrust, it has a substantial impact on power capping above rated. Note that, at cut-out speed, the power for the twist-only case is approximately 22% larger than the case with coupled flapwise and twist deformations, highlighting the importance of considering both components in hydrodynamic analyses, especially when blade deflections are significant.

### 4.3 Net energy calculation

We estimate the average energy yield for the turbine control strategies over a full tidal cycle at two different sites with semi-diurnal tidal patterns. We selected the Pentland Skerries and the Race of Alderney as examples of sites being considered for tidal development. The peak spring and neap tidal flow speeds are presented in Table 4, with data from Black and Veatch (2005). The currents were estimated based on the principal lunar and solar semi-diurnal components $M_{2}$ and $S_{2}$, respectively, defining the flow speed as a time-dependant function:

(8) $$V_{\infty }\left(t\right)=M_{2}\cos\left(\omega_{M2}t\right)+S_{2}\cos\left(\omega_{S2}t\right),$$

where $\omega_{M2}$ and $\omega_{S2}$ are the frequencies of the lunar and solar forces acting on the tides, corresponding to periods of 12.42 and 12 h, respectively. $M_{2}$ and $S_{2}$ are the amplitudes of the tidal speeds, where the peak spring tide flow speed $V_{SP}=M_{2}+S_{2}$ and the peak neap tide flow speed $V_{NP}=M_{2}-S_{2}$.

**Fig. 16:**
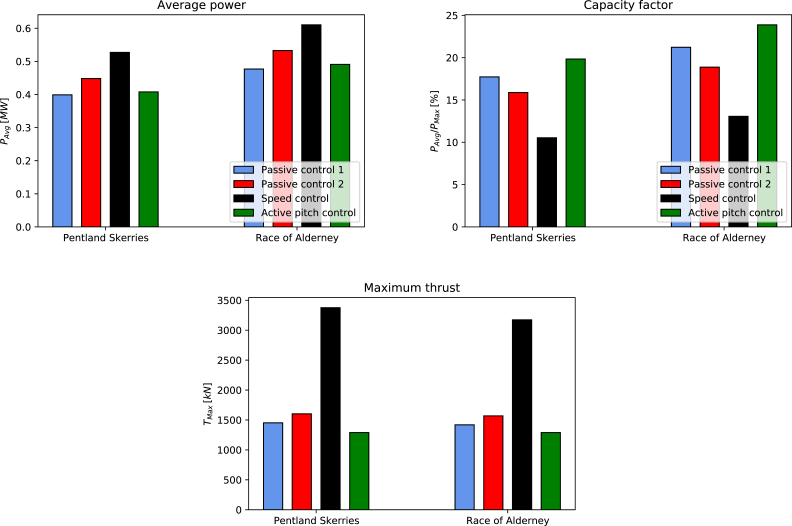
Results comparing the average power, capacity factor and maximum thrust of the four different strategies at the Pentland Skerries and Race of Alderney. (For interpretation of the references to colour in this figure legend, the reader is referred to the web version of this article.)

The average power and maximum thrust through the tidal cycle were calculated under quasi-steady assumptions, and are presented in Fig. 16. The passive control strategies are compared with the speed control strategy defined in Section 4.1, and with an idealised active pitch control case following the speed-controlled rotor with rigid blades below rated, and the power limited to be equal to that at $V_{R}$ for flow speeds above. The maximum thrust is assumed to occur at rated flow speed for the active pitch control case.

The results presented in Fig. 16 show that the speed-controlled strategy with rigid blades has a higher average power, 29% and 24% higher than the active pitch strategy at Pentland Skerries and Race of Alderney, respectively. Nevertheless, the increase in power occurs at the cost of maximum thrust that exceeds the active pitch strategy by 162% and 146%, respectively.

Passive control strategy 1 is the closest strategy in performance to the rotor with active pitch control. It shows an average power production 2% below the idealised active pitch control strategy at the Pentland Skerries, and 3% below at the Alderney Race. The passive control 2 strategy shows an average power that exceeds the active pitch case by 9% and 8% at each site respectively. The closeness of the power production results, despite the passive deformation strategies resulting in sub-optimal performance below rated flow speed, is achieved by compensating for this loss by exceeding the rated power by ca. 12% and 37% for passive control 1 and 2 strategies, respectively, at the cut-out speed.

The capacity factor, defined as the ratio of the average power, $P_{Avg}$, to the maximum power, $P_{Max}$, is also plotted in Fig. 16. This plot highlights the requirement for a larger generator for the passive and speed-controlled strategies compared with the active pitch rotor. However, the passive strategies significantly outperform the speed-controlled rigid rotor, with capacity factors that are 11% (11%) and 20% (21%) lower than the active-pitch rotor for the strategies 1 and 2, respectively, at the Pentland Skerries (Race of Alderney), due to the lower maximum power with passive control. In comparison, the speed-controlled rigid blade rotors show a capacity factor that is 47% (45%) lower than the active pitch rotor at the same site.

The substantial power capping above rated shown by the passive control strategies is linked to a moderate increase in maximum thrust, defined from design, compared to the active pitch strategy. The passive control 1 strategy shows a maximum thrust 11% (9%) higher than the active pitch case, while the passive control 2 shows an excess of 20% (18%) above the idealised active pitch strategy for a rotor located at the Pentland Skerries (Race of Alderney).

**Table 4:** Spring and neap tidal currents at two sites considered for tidal stream turbine deployment.

Site	Mean spring peak $\left(V_{SP}\right)\left[\mathrm{m/s}\right]$	Mean neap peak $\left(V_{NP}\right)\left[\mathrm{m/s}\right]$
Pentland skerries	6.18	2.64
Race of alderney	4.38	2.41

## 5 Conclusions

The results presented in this work highlight important hydrodynamic effects on tidal rotors induced by the flapwise and twist blade deformation components. While the latter seems to be the dominant cause for thrust changes, the flapwise component has a significant impact on integrated power performance that should not be neglected in engineering models and design tools. This work also supports the hypothesis that the hydrodynamics of both components can be regarded as independent from each other for moderate blade deflections. This is, at first, a surprising result, considering that flapwise deformations have an impact on blade loads and performance that cannot be explained through two-dimensional blade-element theory.

The inboard load augmentation and near-tip load shedding, characteristics of flapwise-only cases, were found to be important in cases with coupled deformations. However, the analysis of blade forces revealed that no significant interaction effects between the deformation components occur for moderate blade deflection, and the forces on a blade with coupled deformations can be estimated based on the summation of the load modifications that would arise from flapwise-only and twist-only force distributions.

In terms of the constituent components of engineering models, we observed that the relationship between thrust coefficients and axial induction factors was not significantly impacted by flapwise or twist deformations. Twist deformations were observed to affect the local angle of attack as an apparent modification to the local blade twist angle, but did not affect the underlying lift and drag characteristics along the blade. Therefore the impacts of twist deformations could be included in engineering models by adjusting the blade twist distribution informed by a structural model. Flapwise deformations were found to change the lift and drag characteristics at different spanwise locations along the blade. No significant interactions were observed between the hydrodynamic effects of flapwise and twist deformations, and twist deformations in the presence of flapwise deformations did not induce significant deviations from the lift and drag characteristics corresponding to a flapwise-only case. This suggests that the inclusion of the flapwise deformation hydrodynamics in engineering models could be made through corrections of the polar coefficients.

Despite the changes in polar coefficients caused by the flapwise deformations, which are a consequence of changes in the chordwise pressure distribution, flapwise deformations did not affect significantly the location of the hydrodynamic centre of pressure, which was shown to be dominated by twist and rotational speed (i.e. changes in angle of attack).

Based on the hydrodynamic independence hypothesis for the twist and flapwise deformation, the hydrodynamic performance of turbine blades with passively-controlled load alleviation was investigated to determine the range of flapwise and twist deformations required to satisfy turbine thrust and power objectives.

A parameter space of tip-speed ratio, flapwise and twist deformations was constructed based on the available flapwise-only and twist-only simulations. This parameter space was explored by designing two control paths for passive blade deformation strategies. These strategies showed a substantial power capping above rated flow speed and maximum thrust reductions driven by blade deformations. These results suggest that passive control strategies are a potential alternative for tidal rotors as a replacement for, e.g., active pitch systems. However, an impact on capacity factor is likely to be observed even in cases where the average power production is similar for active and passive control strategies. This is a factor to be considered in cost optimisation to determine the full impact in LCOE.

The passive strategies highlighted the importance of considering both the twist and flapwise deformation hydrodynamics, even for modest blade deflections. While turbine performance is dominated by twist, the flapwise component showed a significant impact on power above rated flow speed, shedding an equivalent of up to 22% of power at cut-out for one of the strategies investigated.

Finally, the independence of the flapwise and twist hydrodynamics for moderate deformations offers potential for the development of improved design methods. Twist deformations, largely reflected in changes in angle of attack, are well-suited for representation in low-cost BEM models. Flapwise deformations, on the contrary, could be included in computations either by using empirical corrections or by performing a reduced set of CFD simulations. This could enable a coupled hydrodynamic and structural design of rotors, incorporating and taking advantage of the hydrodynamics of flapwise deformations on blade loads and power performance.

## CRediT authorship contribution statement

**Federico Zilic de Arcos:** Conceptualization, Methodology, Software, Formal analysis, Investigation, Data curation, Resources, Writing – original draft. **Christopher R. Vogel:** Conceptualization, Methodology, Formal analysis, Resources, Supervision, Writing – review & editing. **Richard H.J. Willden:** Resources, Supervision, Writing – review & editing.

## Declaration of Competing Interest

The authors declare the following financial interests/personal relationships which may be considered as potential competing interests: Federico Zilic de Arcos reports financial support was provided by National Commission for Scientific and Technological Research. Richard Willden reports financial support was provided by Engineering and Physical Sciences Research Council. Christopher Vogel reports financial support was provided by UK Research and Innovation.

## Data Availability

Data will be made available on request.
